# Is there an association between serum 25(OH)D_3_ and mental well-being in patients with type 2 diabetes? Results from a cohort study in primary care

**DOI:** 10.1007/s42000-020-00190-1

**Published:** 2020-05-21

**Authors:** Maria Samefors, Robert Scragg, Fredrik H. Nystrom, Carl Johan Östgren

**Affiliations:** 1 Department of Health, Medicine and Caring Sciences, Linköping University, SE 581 83 Linköping, Sweden; 2 School of Population Health, Faculty of Medical and Health Sciences, University of Auckland, Private Bag 92019, Auckland, New Zealand

**Keywords:** Vitamin D, Type 2 diabetes, Mental health, Vitality

## Abstract

**Purpose:**

There are limited and inconsistent results on the correlation between vitamin D and mental health in patients with type 2 diabetes (T2D). Thus, our aim was to explore the association between vitamin D and mental well-being in a community-based sample of participants with T2D.

**Methods:**

We analyzed serum 25-hydroxyvitamin D_3_ (25(OH)D_3_) in 698 patients with T2D at the baseline examination. The cohort was reinvestigated after 4 years. Data from SF-36 questionnaires measuring vitality and mental health at baseline and after 4 years were used for analyses.

**Results:**

Serum 25(OH)D_3_ was inversely associated with poor mental health at baseline (odds ratio (OR) for 10 nmol/l increase in 25(OH)D_3_, 0.90 (95% confidence interval (CI) 0.83–0.96, *p* = 0.003)) but not at follow-up (*p* > 0.05). Serum 25(OH)D_3_ was not associated with vitality at baseline (*p* > 0.05). At follow-up, there was an inverse association between 25(OH)D_3_ and low vitality (OR for 10 nmol/l increase in 25(OH)D_3_, 0.89 (95% CI 0.82–0.97, *p* = 0.009)) but not after adjustment.

**Conclusion:**

We found an inverse association between 25(OH)D_3_ and mental health in patients with T2D at baseline. We found no association between 25(OH)D_3_ and vitality after adjustment. Future studies are needed to determine the association between vitamin D and mental well-being in patients with T2D.

**Electronic supplementary material:**

The online version of this article (10.1007/s42000-020-00190-1) contains supplementary material, which is available to authorized users.

## Introduction

The prevalence of depression in patients with diabetes is twice that of those without diabetes and even more elevated in women than in men [1, 2]. Diabetes may increase the risk of subsequent depression and vice versa [3]. A systematic review of the bidirectional relationship has discussed the potential mechanisms. For individuals with diabetes, these include the psychosocial burden of having a chronic disease, the need for a high level of self-care including blood glucose monitoring, management of medication and strict nutrition, and the biochemical changes associated with diabetes—all of which may lead to depression. For persons with depressive disorders, behavioral factors such as diet, exercising, and smoking habits associated with obesity and insulin resistance, poor self-care behavior, and biochemical changes, including increased hypothalamic-pituitary-adrenocortical axis activity and sympathetic nervous system activity resulting in elevated cortisol levels, together with increased synthesis of pro-inflammatory cytokines, may lead to diabetes [4]. However, a full understanding of the correlation between type 2 diabetes (T2D) and depression is yet lacking. It would appear that important knowledge concerning certain biological factors is missing that would help to explain this association.

Several previous studies have demonstrated an association between low levels of vitamin D and increased risk of impaired glucose tolerance, insulin resistance, and incidence of T2D [5, 6]. Furthermore, systematic reviews and meta-analyses have shown an association between low vitamin D levels and risk of depression [7, 8], which has been replicated in more recent studies [9–14]. The majority of previous studies have been cross-sectional, while in most [10, 13, 15, 16], but not all [17], longitudinal studies, an association between low baseline blood 25(OH)D (25-hydroxyvitamin D) and development of depressive symptoms has been found. The association between vitamin D and mood disorders has been explored in patients with cardiovascular disease [16], obesity [18], and post-stroke [14]. To the best of our knowledge, only two studies of patients with T2D concerning the association between vitamin D and depression have been published, these presenting contradictory results. In the first study, serum 25(OH)D level was not a mediatory factor in the association between impaired glucose tolerance/impaired fasting glucose or T2D and depressive symptoms [19]; however, this study was small with only 104 T2D patients. In the second study, an inverse association between serum 25(OH)D levels and depression was found in patients with T2D [20]; however, since the participants were recruited from hospital-based clinics, they may not be representative of all individuals with T2D. Another study examined the effects of vitamin D supplementation on dimensions of health-related quality of life in patients with T2D [21].

Given the high prevalence of T2D, depression, and vitamin D deficiency, an association between vitamin D deficiency and mental well-being in T2D patients could have potential public health implications. Thus, we explored the association between data from patient questionnaires measuring mental well-being in terms of vitality and mental health at baseline and after 4 years together with baseline levels of vitamin D in a community-based sample of participants with T2D in primary care.

## Methods

### Participants

We analyzed data from T2D patients aged 55–66 years (*n* = 761) who participated in a prospective observational community-based cohort study: CARDIPP (Risk factors in Patients with Diabetes—a Prospective study in Primary Care), which has been described previously [22–24]. The participants were consecutively recruited from 22 primary health care centers in southern Sweden. The baseline examination was performed between 2005 and 2008 and all participants were invited to a reinvestigation after 4 years.

### General questionnaire

The study participants filled out a questionnaire about their marital status, occupation, well-being, and lifestyle factors, which included exercising, smoking, and drinking habits at baseline. A standardized anamnesis was taken that included ongoing medication inclusive of vitamin D and other supplements.

### Questions on mental well-being

The short form health survey SF-36 was used to measure mental well-being in terms of vitality and mental health. SF-36 is a widely used, standardized questionnaire derived from the Medical Outcomes study in the 1980s measuring self-reported physical and mental health status [25]. The study participants were asked to answer the questions regarding the health concepts vitality and mental health depicted in Table 1. The items were assessed with a range from 1 to 6, i.e., 1 = all of the time, 2 = most of the time, 3 = a good bit of the time, 4 = some of the time, 5 = a little of the time, and 6 = none of the time. According to a standardized scoring protocol [26], the responses to each item were scored and summed, and the scores for each health concept were expressed as a score between 0 and 100, where a higher score represents better self-perceived health. An evaluation of SF-36 regarding data quality, scaling assumptions, reliability, and construct validity has been made in Sweden with reliable results [27].

**Table 1:** Questions on vitality and mental health from the short form health survey SF-36

How much of the time during the past 4 weeks:	
1. Did you feel full of pep?	
2. Have you been a very nervous person?	
3. Have you felt so down in the dumps that nothing could cheer you up?	
4. Have you felt calm and peaceful?	
5. Did you have a lot of energy?	
6. Have you felt downhearted and blue?	
7. Did you feel worn out?	
8. Have you been a happy person?	
9. Did you feel tired?	

The health concept of vitality was measured using questions 1, 5, 7, and 9 and of mental health using questions 2, 3, 4, 6, and 8

### Physical measurements

Nurses dedicated to treatment of diabetes measured body weight to the nearest 0.1 kg and height to the nearest centimeter with the patients wearing light clothing and without shoes, using a standardized protocol and standardized equipment in the local laboratory of the health care centers. BMI (body mass index) was calculated from the weight in kilograms divided by the square of the height in meters. The average of three manually measured sitting blood pressures with 1 min between each measurement was used for analysis [28].

### Laboratory methods

A venous blood sample was taken from participants at baseline in the fasting state and was used for immediate routine laboratory analyses and for biobanking of plasma/serum/urine aliquots frozen and stored for later analyses. From the frozen samples, serum 25(OH)D_3_ (25-hydroxyvitamin D_3_) was analyzed using chemoluminiscense on a Cobas e602 unit (Roche Diagnostics Scandinavia AB, Bromma, Sweden). Details of the laboratory methods have been described previously [24].

### Statistical analyses

For the statistical analyses, we used IBM SPSS Statistics 23 (International Business Machines Corporation, New York, USA). Non-parametric tests were used, as the quantitative variables were not normally distributed. Median was used as average value and interquartile range (IQR) as a measure of statistical dispersion. The Mann-Whitney *U* test and Kruskal-Wallis H test were used to compare median levels of continuous variables between groups. Regarding the results of SF-36, we also presented mean and standard deviation (SD), and the one sample *t* test was used to compare our results with the mean values of the Swedish population. The chi-square test was used to investigate the associations between categorical data. We analyzed Spearman’s correlation coefficients to assess associations between serum 25(OH)D_3_ and key continuous variables, vitality, and mental health. To calculate OR (odds ratio) for vitality and mental health associated with serum 25(OH)D_3_, we used binary logistic regression analyses. The median scores were used to dichotomize the concepts of vitality and mental health from the SF-36. All covariates used in all the logistic regression models were measured at baseline. Statistical significance was defined as *p* < 0.05. We categorized April–September as summer, and January–March and October–December as winter in order to adjust for the season when the blood samples for vitamin D were drawn.

## Results

CARDIPP had 761 participants. In total, 63 subjects were excluded because of vitamin D supplementation (*n* = 20) or missing data on vitamin D status (*n* = 44). One subject had both vitamin D supplementation and missing data on vitamin D status. Of the 698 subjects remaining for further analyses, 463 (66%) were male and 235 (34%) female.

### Serum 25(OH)D_3_ and baseline measurements

The range of serum 25(OH)D_3_ was 7.5–164.9 nmol/l and the median (IQR) 25(OH)D_3_ concentration was 47.5 (26.3) nmol/l. The baseline characteristics of the study population, categorized by serum 25(OH)D_3_ quartiles, are shown in Table 2. There was no difference in marital/cohabiting status between the quartiles. No difference in occupation regarding professional work, unemployment, long-term sick leave, or early retirement was seen, but there was a difference between the quartiles regarding retirement due to old age with a higher proportion of old-age retirees in the highest 25(OH)D_3_ quartile (*p* = 0.030). There was a variation between the vitamin D quartiles in self-reported exercising habits with the highest proportions of participants categorized as sedentary or doing mild exercise being in the quartile with the lowest vitamin D levels (*p* = 0.005). Regarding alcohol use and smoking habits, no variation was seen. The baseline characteristics regarding age, gender, anthropometric measurements, and laboratory data have been described previously [24]. Spearman’s correlation coefficients between 25(OH)D_3_ and key continuous variables are presented in Supplementary Table 1.

**Table 2:** Baseline characteristics according to serum 25(OH)D_3_ quartiles

	Serum 25(OH)D_3_ quartile				*p* value
	1	2	3	4	
Number	174	175	175	174	
Range 25(OH)D_3_ (nmol/l)	7.5–35.5	35.5–47.5	47.5–61.8	61.8–164.9	
Male	63%	64%	67%	71%	0.38
Age (years)	60.0 (5.0)	61.0 (5.0)	61.0 (5.0)	62.0 (5.0)	0.006*
Married or cohabiting	77%	84%	80%	83%	0.33
Occupation^a^					
Professional work	61%	62%	66%	60%	0.76
Unemployment	4%	5%	5%	5%	0.99
Sick leave > 3 months	5%	2%	4%	3%	0.47
Early retirement	32%	28%	24%	24%	0.30
Old age pension	4%	9%	6%	13%	0.030*
Exercise					0.005*
Sedentary	18%	10%	14%	10%	
Mild	66%	57%	59%	57%	
Moderate	8%	25%	19%	22%	
Regular	9%	8%	9%	12%	
Smoking					.060
Current	27%	16%	18%	16%	
Previous	42%	51%	47%	56%	
None	32%	33%	35%	28%	
Alcohol use					0.17
> 4 times a week	3%	3%	3%	6%	
2–3 times a week	15%	12%	14%	19%	
2–4 times a month	32%	41%	43%	41%	
< Once a month	28%	28%	26%	23%	
Never	21%	16%	15%	11%	
BMI (kg/m^2^)	30.5 (6.1)	29.8 (6.3)	29.4 (5.8)	29.0 (5.7)	0.001*
Systolic blood pressure (mmHg)	137.7 (21.3)	140.0 (21.3)	133.3 (19.0)	136.7 (22.4)	0.004*
Diastolic blood pressure (mmHg)	79.3 (14.8)	80.0 (11.2)	78.7 (13.7)	81.2 (14.8)	0.12
Calcium, albumin-corrected (mmol/l)	2.3 (0.1)	2.3 (0.1)	2.3 (0.1)	2.3 (0.1)	0.40
PTH (pg/ml)	47.9 (22.1)	45.4 (18.2)	43.1 (16.3)	40.1 (15.6)	p < 0.001*
HbA1c (mmol/mol)	53.1 (16.7)	51.0 (13.1)	50.0 (13.6)	50.0 (14.9)	0.003*
Creatinine (μmol/l)	83.0 (19.0)	86.0 (22.0)	84.0 (22.8)	88.0 (26.0)	0.013*
GFR (ml/min/1.73m^2^)	79.9 (22.9)	77.1 (24.1)	77.6 (25.3)	74.1 (23.0)	0.039*
Duration of diabetes (years)	7.0 (8.0)	6.0 (9.0)	5.0 (6.0)	6.0 (6.0)	0.018*

Data are presented as medians and interquartile range or as percentages. The Kruskal-Wallis H test was used to compare median levels between groups in quantitative data. The chi-square test was used to investigate associations between categorical data. A *p* value of < 0.05 was considered significant (*)

*25(OH)D*_*3*_, 25-hydroxyvitamin D_3_; *BMI*, body mass index; *PTH*, parathyroid hormone; *HbA1c*, hemoglobin A1c; *GFR*, glomerular filtration rate

^a^Based on the instructions given for the item of occupation, some participants filled out more than one category, which explains why the sum of occupation for each quartile exceeds 100%

### Mental well-being from SF-36 and follow-up

Data on vitality and mental health according to SF-36 was available for 98% (*n* = 687 for vitality/*n* = 686 for mental health) of the study participants at baseline and for 67% (*n* = 469 for vitality/*n* = 467 for mental health) at the follow-up after 4 years. In total, the median (IQR) score for vitality was 70 (30), and the mean (SD) score was 63.7 (23.2) at baseline. At follow-up, the median (IQR) score for vitality was 70 (35), and the mean (SD) score was 64.8 (22.5). The median (IQR) score for mental health was 84 (20) both at baseline and at follow-up. The mean (SD) score was 80.4 (17.5) at baseline and 80.4 (17.1) at follow-up. The Spearman’s correlation coefficients between 25(OH)D_3_ and vitality and mental health scores are presented in Supplementary Table 2.

Although the average vitality and mental health scores did not change over the follow-up period, there were individual differences. The number of study participants with deteriorated scores in vitality respective to mental health at follow-up compared with baseline was 188. The number of study participants with deteriorated scores of both health concepts at follow-up was 109. There were no differences between the participants who showed deterioration in vitality and/or mental health at follow-up and the participants who had unchanged/improved scores regarding the baseline characteristics except for occupational status. This is further elaborated in Supplementary Table 3.

The study participants who did not attend the follow-up (*n* = 229) more often were unmarried/living alone, sedentary, current smokers, and obese compared with participants who attended the follow-up (*n* = 469). Furthermore, the non-attendees at follow-up had longer diabetes duration but lower diastolic blood pressure and lower levels of PTH, 25(OH)D_3_, and creatinine (*p* < 0.05). The median (IQR) baseline 25(OH)D_3_ for those who attended the reinvestigation was 49.0 (26.3) nmol/l compared with 44.7 (26.1) nmol/l for those who did not attend the reinvestigation (*p* = 0.006). This is further elaborated in Supplementary Table 4.

### Adjusted associations between 25(OH)D_3_ and mental well-being

Table 3 shows the ORs of vitality and mental health for a 10 nmol/l increase in baseline serum 25(OH)D_3_ in five models. Baseline serum 25(OH)D_3_ was inversely associated with poor mental health in all models in the baseline analyses (*p* < 0.05) but not in the 4-year follow-up analyses. Serum 25(OH)D_3_ was not associated with vitality at baseline. At follow-up, baseline serum 25(OH)D_3_ was associated inversely with low vitality in three models but not when adjusting for diabetes-related data or lifestyle factors.

**Table 3:** Odds ratios of mental well-being at baseline and at 4-year follow-up associated with a 10 nmol/l increase in baseline 25(OH)D_3_

	At baseline (OR (95% CI), *p* value)	At 4-year follow-up (OR (95% CI), *p* value)
Vitality (score below median)	*n* = 687	*n* = 469
Unadjusted	0.94 (0.87–1.00), *p* = 0.065	0.89 (0.82–0.97), *p* = 0.009*
Adjusted for age and sex	0.95 (0.89–1.02), *p* = 0.19	0.91 (0.83–1.00), *p* = 0.039*
Adjusted for age, sex, season, PTH, and albumin-corrected calcium	0.96 (0.89–1.03), *p* = 0.26	0.89 (0.81–0.98), *p* = 0.014*
Adjusted for age, sex, BMI, HbA1c, and duration of diabetes	0.97 (0.90–1.05), *p* = 0.42	0.94 (0.86–1.04), *p* = 0.21
Adjusted for age, sex, BMI, marital status, physical activity, smoking, and alcohol habits	0.96 (0.89–1.04), *p* = 0.33	0.94 (0.86–1.04), *p* = 0.24
Mental health (score below median)	*n* = 686	*n* = 467
Unadjusted	0.90 (0.83–0.96), *p* = 0.003*	0.94 (0.87–1.03), *p* = 0.18
Adjusted for age and sex	0.91 (0.84–0.98), *p* = 0.010*	0.96 (0.88–1.04), *p* = 0.32
Adjusted for age, sex, season, PTH, and albumin-corrected calcium	0.91 (0.84–0.98), *p* = 0.017*	0.94 (0.86–1.03), *p* = 0.21
Adjusted for age, sex, BMI, HbA1c, and duration of diabetes	0.92 (0.85–1.00), *p* = 0.046*	0.98 (0.90–1.08), *p* = 0.71
Adjusted for age, sex, BMI, marital status, physical activity, smoking, and alcohol habits	0.91 (0.84–0.99), *p* = 0.021*	0.98 (0.89–1.07), *p* = 0.65

A *p* value of < 0.05 was considered significant (*)

*25(OH)D*_*3*_, 25-hydroxyvitamin D_3_; *OR*, odds ratio; *CI*, confidence interval; *PTH*, parathyroid hormone; *BMI*, body mass index; *HbA1c*, hemoglobin A1c

## Discussion

In this community-based cohort study with T2D patients, we found an inverse association between vitamin D and poor mental health at baseline, and the association remained significant when adjusting for confounders such as season, clinical data, and lifestyle factors. This finding is consistent with one [20] of the two studies confined to populations with diabetes and with the majority of previous studies in non-diabetic populations [7–9, 11, 12, 14]; however, it is not consistent with most of the cohort studies reporting an association between low baseline vitamin D levels and future development of depressive symptoms [10, 13, 15, 16], as we did not find any association at follow-up.

For vitality, no association with vitamin D was found at baseline. At follow-up, the significant association first seen disappeared when clinical data regarding diabetes status and lifestyle factors were added into the analyses. Our results indicate that other factors have a larger impact on vitality than vitamin D in patients with T2D. The participants in our study had a significantly lower mean score for vitality compared with the Swedish general population (mean score 68.8) [27] both at baseline (*p* < 0.001) and at follow-up (*p* < 0.001), which is not surprising as T2D is a chronic disease. In contrast, no difference was seen for mental health (*p* > 0.05) compared with the Swedish general population (mean score 80.9) [27].

Our study is different compared with previous studies, as the outcomes in our study (dimensions of mental well-being) differ compared with those of previous studies (depressive disorders/symptoms), which may weaken the association. In our study, we used self-reported answers in questionnaires to measure mental well-being. In a previous meta-analysis, the association between vitamin D status and risk of depression was strongest in cross-sectional studies using a structured clinical diagnostic interview or clinical diagnosis to identify depression instead of a self-reported symptom scale [7].

The baseline characteristics of the study population have partly been described previously in a study exploring the association between vitamin D and cardiovascular morbidity and mortality [24]. However, the current study targets the issue concerning the association between vitamin D levels and mental well-being in the study cohort. As the previous research on vitamin D and mental health in patients with T2D is limited and inconsistent, our results expand the current knowledge on the association between vitamin D and mental well-being.

Different mechanisms through which vitamin D may influence brain functions and lead to depressive symptoms have been proposed. VDR (vitamin D receptor) [29] and the enzyme 1-alpha-hydroxylase, required for production of the active form of vitamin D, 1,25(OH)_2_D_3_ (1,25-dihydroxyvitamin D_3_) [30], are distributed throughout the human brain, suggesting possible autocrine/paracrine effects of vitamin D in the brain. An interaction between vitamin D and glucocorticoids in the hippocampus has been found [31]. Vitamin D is involved in several different brain processes including regulation of neurotrophic factors, neuroplasticity, and neuroprotection [32]. Vitamin D also has a modulatory effect on pro-inflammatory cytokines and downregulates inflammatory mediators, which is a possible link between vitamin D deficiency and depression [33]. Vitamin D might also have direct neuroregulatory effects, as genetic variance in the VDR gene influences cognitive functions and the prevalence of depressive symptoms in older individuals [34]. It is also plausible that depressive disorders lead to a poor diet and fewer outdoor activities, resulting in less sunlight exposure and lower 25(OH)D_3_ concentrations, i.e., low vitamin D levels are not the cause but the effect of the depression.

### Strengths and limitations

The main strength of this study is that it is a community-based study among patients with T2D, and the results could be generalized to all patients with T2D. Other strengths include the prospective study design and the fact that several confounders could be integrated in the analyses. This study also has some weaknesses. We had no data on the medical history of depression or other prior diagnoses of affective disorders, nor any data on antidepressant medication. We had to rely on a single 25(OH)D_3_ value drawn at baseline for calculations of the association between vitamin D and the mental well-being both at baseline and follow-up, which is a weakness of the follow-up analyses. One-third of the study population was lost to follow-up after 4 years. There are several differences between those of the groups who did not attend the reinvestigation, including significantly lower baseline vitamin D values, compared with those who did attend the reinvestigation. This potential selection bias might weaken the association between vitamin D and mental health and vitality at follow-up. There is a discrepancy between the size of the associations between serum 25(OH)D_3_ and vitality and mental health in Spearman’s correlations and the size of the results of the logistic regression analyses, which is why we cannot rule out moderation of an unrecognized factor or a non-linear association. Due to the observational study design, we cannot preclude residual confounding and cannot draw conclusions about causality.

In summary, in this study with middle-aged patients with T2D, we found an inverse association between serum 25(OH)D_3_ and poor mental health at baseline but not at follow-up. We found no association between serum 25(OH)D_3_ and vitality at baseline, while at follow-up, the association first seen disappeared after controlling for confounding factors. Future prospective studies and clinical trials are warranted to better define the association between vitamin D and mental well-being in patients with T2D.

## Electronic supplementary material

ESM 1 (DOCX 29.8  kb)

## Data Availability

The dataset generated and analyzed during the current study is available from the corresponding author on reasonable request.

## References

[1] Anderson RJ, Freedland KE, Clouse RE, Lustman PJ (2001). The prevalence of comorbid depression in adults with diabetes: a meta-analysis. Diabetes Care.

[2] Roy T, Lloyd CE (2012) Epidemiology of depression and diabetes: a systematic review. J Affect Disord 142 Suppl:S8–21. 10.1016/s0165-0327(12)70004-6 10.1016/S0165-0327(12)70004-6 23062861

[3] Mezuk B, Eaton WW, Albrecht S, Golden SH (2008). Depression and type 2 diabetes over the lifespan: a meta-analysis. Diabetes Care.

[4] Renn BN, Feliciano L, Segal DL (2011). The bidirectional relationship of depression and diabetes: a systematic review. Clin Psychol Rev.

[5] Song Y, Wang L, Pittas AG, Del Gobbo LC, Zhang C, Manson JE, Hu FB (2013). Blood 25-hydroxy vitamin D levels and incident type 2 diabetes: a meta-analysis of prospective studies. Diabetes Care.

[6] Khan H, Kunutsor S, Franco OH, Chowdhury R (2013). Vitamin D, type 2 diabetes and other metabolic outcomes: a systematic review and meta-analysis of prospective studies. Proc Nutr Soc.

[7] Ju SY, Lee YJ, Jeong SN (2013). Serum 25-hydroxyvitamin D levels and the risk of depression: a systematic review and meta-analysis. J Nutr Health Aging.

[8] Anglin RE, Samaan Z, Walter SD, McDonald SD (2013). Vitamin D deficiency and depression in adults: systematic review and meta-analysis. Br J Psychiatry J Ment Sci.

[9] Jaaskelainen T, Knekt P, Suvisaari J, Mannisto S, Partonen T, Saaksjarvi K, Kaartinen NE, Kanerva N, Lindfors O (2015). Higher serum 25-hydroxyvitamin D concentrations are related to a reduced risk of depression. Br J Nutr.

[10] Milaneschi Y, Hoogendijk W, Lips P, Heijboer AC, Schoevers R, van Hemert AM, Beekman AT, Smit JH, Penninx BW (2014). The association between low vitamin D and depressive disorders. Mol Psychiatry.

[11] Chung HK, Cho Y, Choi S, Shin MJ (2014). The association between serum 25-hydroxyvitamin D concentrations and depressive symptoms in Korean adults: findings from the fifth Korea National Health and Nutrition Examination Survey 2010. PLoS One.

[12] Mizoue T, Kochi T, Akter S, Eguchi M, Kurotani K, Tsuruoka H, Kuwahara K, Ito R, Kabe I, Nanri A (2015). Low serum 25-hydroxyvitamin D concentrations are associated with increased likelihood of having depressive symptoms among Japanese workers. J Nutr.

[13] Brouwer-Brolsma EM, Dhonukshe-Rutten RA, van Wijngaarden JP, van der Zwaluw NL, Sohl E, In't Veld PH, van Dijk SC, Swart KM, Enneman AW, Ham AC, van Schoor NM, van der Velde N, Uitterlinden AG, Lips P, Feskens EJ, de Groot LC (2015) Low vitamin D status is associated with more depressive symptoms in Dutch older adults. Eur J Nutr. 10.1007/s00394-015-0970-6 10.1007/s00394-015-0970-6 PMC4875055 26141257

[14] Yue W, Xiang L, Zhang YJ, Ji Y, Li X (2014). Association of serum 25-hydroxyvitamin D with symptoms of depression after 6 months in stroke patients. Neurochem Res.

[15] Milaneschi Y, Shardell M, Corsi AM, Vazzana R, Bandinelli S, Guralnik JM, Ferrucci L (2010). Serum 25-hydroxyvitamin D and depressive symptoms in older women and men. J Clin Endocrinol Metab.

[16] May HT, Bair TL, Lappe DL, Anderson JL, Horne BD, Carlquist JF, Muhlestein JB (2010). Association of vitamin D levels with incident depression among a general cardiovascular population. Am Heart J.

[17] Chan R, Chan D, Woo J, Ohlsson C, Mellstrom D, Kwok T, Leung P (2011). Association between serum 25-hydroxyvitamin D and psychological health in older Chinese men in a cohort study. J Affect Disord.

[18] Jorde R, Sneve M, Figenschau Y, Svartberg J, Waterloo K (2008). Effects of vitamin D supplementation on symptoms of depression in overweight and obese subjects: randomized double blind trial. J Intern Med.

[19] Westra S, Simsek S, Rutters F, Krul-Poel YM, Stehouwer CD, Dekker JM, Pouwer F (2017). Low vitamin D levels are not a contributing factor to higher prevalence of depressive symptoms in people with type 2 diabetes mellitus: the Hoorn study. Diabet Med.

[20] Wang Y, Yang H, Meng P, Han Y (2017). Association between low serum 25-hydroxyvitamin D and depression in a large sample of Chinese patients with type 2 diabetes mellitus. J Affect Disord.

[21] Westra S, Krul-Poel YH, van Wijland HJ, Ter Wee MM, Stam F, Lips P, Pouwer F, Simsek S (2016). Effect of vitamin D supplementation on health status in non-vitamin D deficient people with type 2 diabetes mellitus. Endocr Connect.

[22] Blomstrand P, Engvall M, Festin K, Lindstrom T, Lanne T, Maret E, Nystrom FH, Maret-Ouda J, Ostgren CJ, Engvall J (2015) Left ventricular diastolic function, assessed by echocardiography and tissue Doppler imaging, is a strong predictor of cardiovascular events, superior to global left ventricular longitudinal strain, in patients with type 2 diabetes. Eur Heart J Cardiovasc Imaging. 10.1093/ehjci/jev027 10.1093/ehjci/jev027 25750201

[23] Spangeus A, Wijkman M, Lindstrom T, Engvall JE, Ostgren CJ, Nystrom FH, Lanne T (2013). Toe brachial index in middle aged patients with diabetes mellitus type 2: not just a peripheral issue. Diabetes Res Clin Pract.

[24] Samefors M, Scragg R, Lanne T, Nystrom FH, Ostgren CJ (2017). Association between serum 25(OH)D3 and cardiovascular morbidity and mortality in people with type 2 diabetes: a community-based cohort study. Diabet Med.

[25] Ware JE, Sherbourne CD (1992). The MOS 36-item short-form health survey (SF-36). I. Conceptual framework and item selection. Med Care.

[26] Sullivan M, Karlsson J, Taft C (2002) SF-36 health survey: Swedish manual and interpretation guide. 2nd edition edn. Sahlgrenska University Hospital, Göteborg

[27] Sullivan M, Karlsson J, Ware JE (1995). The Swedish SF-36 health survey--I. evaluation of data quality, scaling assumptions, reliability and construct validity across general populations in Sweden. Soc Sci Med.

[28] Wijkman M, Lanne T, Ostgren CJ, Nystrom FH (2016). Diastolic orthostatic hypertension and cardiovascular prognosis in type 2 diabetes: a prospective cohort study. Cardiovasc Diabetol.

[29] Eyles DW, Smith S, Kinobe R, Hewison M, McGrath JJ (2005). Distribution of the vitamin D receptor and 1 alpha-hydroxylase in human brain. J Chem Neuroanat.

[30] Zehnder D, Bland R, Williams MC, McNinch RW, Howie AJ, Stewart PM, Hewison M (2001). Extrarenal expression of 25-hydroxyvitamin d(3)-1 alpha-hydroxylase. J Clin Endocrinol Metab.

[31] Obradovic D, Gronemeyer H, Lutz B, Rein T (2006). Cross-talk of vitamin D and glucocorticoids in hippocampal cells. J Neurochem.

[32] Fernandes de Abreu DA, Eyles D, Feron F (2009). Vitamin D, a neuro-immunomodulator: implications for neurodegenerative and autoimmune diseases. Psychoneuroendocrinology.

[33] McCann JC, Ames BN (2008). Is there convincing biological or behavioral evidence linking vitamin D deficiency to brain dysfunction?. FASEB J.

[34] Kuningas M, Mooijaart SP, Jolles J, Slagboom PE, Westendorp RG, van Heemst D (2009). VDR gene variants associate with cognitive function and depressive symptoms in old age. Neurobiol Aging.

