# Supplementary material for: Is there an association between serum 25(OH)D3 and mental well-being in patients with type 2 diabetes? Results from a cohort study in primary care
Source: Hormones (Athens). 2020 May 21;19(3):361–7. doi: 10.1007/s42000-020-00190-1 (PMC7426318; doi:10.1007/s42000-020-00190-1)
Supplement: Supplementary file 1 — (DOCX 29.8 kb) [file 42000_2020_190_MOESM1_ESM.docx]

### Supplementary Material:

**Is there an association between serum 25(OH)D_3_ and mental well-being in patients with type 2 diabetes? Results from a cohort study in primary care**

| **Supplementary Table 1: Spearman´s correlation coefficients between 25(OH)D_3_ and key continuous variables** | | | | | | | | |
| --- | --- | --- | --- | --- | --- | --- | --- | --- |
|  |  | 25(OH)D3 | Age | BMI | Diabdur | PTH | Alb.corr Calcium | HbA1c |
| Age | Correlation Coefficient | 0.126 |  |  |  |  |  |  |
|  | p-value | 0.001* |  |  |  |  |  |  |
| BMI | Correlation Coefficient | -0.160 | -0.001 |  |  |  |  |  |
|  | p-value | <0.001* | 0.981 |  |  |  |  |  |
| Diabdur | Correlation Coefficient | -0.065 | 0.128 | 0.013 |  |  |  |  |
|  | p-value | 0.098 | 0.001* | 0.734 |  |  |  |  |
| PTH | Correlation Coefficient | -0.191 | -0.068 | 0.095 | -0.065 |  |  |  |
|  | p-value | <0.001* | 0.079 | 0.013* | 0.102 |  |  |  |
| Alb.corr. Calcium | Correlation Coefficient | 0.033 | 0.086 | 0.154 | 0.029 | -0.133 |  |  |
|  | p-value | 0.390 | 0.024* | <0.001* | 0.462 | 0.001* |  |  |
| HbA1c | Correlation Coefficient | -0.119 | -0.029 | 0.097 | 0.321 | -0.019 | 0.065 |  |
|  | p-value | 0.002* | 0.455 | 0.011* | <0.001* | 0.615 | 0.088 |  |
| A p-value of <0.05 was considered significant (*). | | | | | | | | |

25(OH)D_3_, 25-hydroxyvitamin D_3_; BMI, body mass index; Diabdur, duration of diabetes; PTH, parathyroid hormone; Alb.corr Calcium, albumin-corrected Calcium; HbA1c, hemoglobin A1c

**Supplementary Table 2: Spearman´s correlation coefficients between 25(OH)D_3_ and vitality and mental health scores**

|  | | | | | | |
| --- | --- | --- | --- | --- | --- | --- |
|  |  | 25(OH)D3 | Vitality | Mental health | Vitality at follow-up | Mental health at follow-up |
| Vitality | Correlation Coefficient | 0.100 |  |  |  |  |
|  | p-value | 0.009* |  |  |  |  |
| Mental health | Correlation Coefficient | 0.121 | 0.674 |  |  |  |
|  | p-value | 0.001* | <0.001* |  |  |  |
| Vitality at follow-up | Correlation Coefficient | 0.164 | 0.666 | 0.481 |  |  |
|  | p-value | <0.001* | <0.001* | <0.001* |  |  |
| Mental health at follow-up | Correlation Coefficient | 0.063 | 0.526 | 0.574 | 0.692 |  |
|  | p-value | 0.171 | <0.001* | <0.001* | <0.001* |  |
| A p-value of <0.05 was considered significant (*). | | | | | | |

25(OH)D_3_, 25-hydroxyvitamin D_3_

**Supplementary Table 3: Significant differences between the participants who showed deterioration in vitality and/or mental health scores in SF-36 and the participants who had unchanged/ improved scores regarding occupational status**

|  | Deterioration in  VT + MH | No deterioration in  VT + MH | p-value |
| --- | --- | --- | --- |
| Unemployment | 8% | 3% | p=0.049 ^a^ |
| Old age pension | 15% | 8% | p=0.033 |
|  |  |  |  |
|  | Deterioration in MH | No deterioration in  MH |  |
| Early retirement | 30% | 22% | p=0.039 |
|  |  |  |  |
|  | Deterioration in  VT | No deterioration in  VT |  |
| Unemployment | 8% | 2% | p=0.002 |
|  |  |  |  |

The Chi-square test was used to investigate associations between categorical data.

^a^ Fischer´s exact test was used when more than 20% of the cells had an expected frequency below 5.

VT, vitality; MH, mental health

**Supplementary Table 4: Comparison of the study participants who attended the follow-up with the study participants who did not attend the follow-up after 4 years on demographic, clinical and laboratory variables at baseline**

|  |  | | |
| --- | --- | --- | --- |
|  | Study participants who attended follow-up | Study participants who not attended follow-up | p-value |
| Number | 469 | 229 |  |
| Male | 68 % | 62 % | p=0.09 |
| Age (years) | 61.0 (5.0) | 61.0 (5.0) | p=0.05 |
| Married or cohabiting | 84 % | 74 % | p=0.003* |
| Occupation^a^ |  |  |  |
| Professional work | 64 % | 59 % | p=0.29 |
| Unemployment | 4 % | 6 % | p=0.40 |
| Sick leave >3 months | 4 % | 3 % | p=0.54 |
| Early retirement | 25 % | 32 % | p=0.06 |
| Old age pension | 9 % | 5 % | p=0.07 |
| Exercise |  |  | p=0.009* |
| Sedentary | 10 % | 18 % |  |
| Mild | 59 % | 60 % |  |
| Moderate | 20 % | 15 % |  |
| Regular | 11 % | 7% |  |
| Smoking |  |  | p<0.001* |
| Current | 15 % | 28 % |  |
| Previous | 52 % | 42 % |  |
| Non- | 33 % | 29 % |  |
| Alcohol use |  |  | p=0.38 |
| => 4 times a week | 4 % | 3 % |  |
| 2-3 times a week | 16 % | 12 % |  |
| 2-4 times a month | 38 % | 41 % |  |
| =< once a month | 27 % | 25 % |  |
| Never | 14 % | 19 % |  |
| BMI (kg/m^2^) | 29.4 (5.7) | 30.4 (6.6) | p=0.003* |
| Systolic blood pressure (mmHg) | 137.3 (20.7) | 135.3 (23.8) | p=0.63 |
| Diastolic blood pressure (mmHg) | 80.0 (12.7) | 78.7 (15.8) | p=0.036* |
| Calcium, albumin-corrected (mmol/l) | 2.3 (0.1) | 2.3 (0.1) | p=0.99 |
| PTH (pg/ml) | 44.5 (18.4) | 42.7 (18.5) | p=0.023* |
| 25(OH)D_3_ (nmol/l) | 49.0 (26.3) | 44.7 (26.1) | p=0.006* |
| HbA1c (mmol/mol) | 50.0 (13.6) | 52.1 (16.2) | p=0.188 |
| Creatinine (µmol/l) | 86.0 (22.0) | 83.0 (19.5) | p=0.033* |
| GFR (ml/min/1.73m^2^) | 76.5 (23.5) | 78.2 (23.4) | p=0.25 |
| Duration of diabetes (years) | 5.0 (6.8) | 7.0 (9.0) | p=0.008* |
| Data are presented as medians and interquartile range or as percentages. The Mann-Whitney U test was used to compare median levels of continuous variables between groups. The Chi-square test was used to investigate associations between categorical data. A p-value of <0.05 was considered significant (*).  BMI, body mass index; PTH, parathyroid hormone; 25(OH)D_3_, 25-hydroxyvitamin D_3_; HbA1c, hemoglobin A1c; GFR, glomerular filtration rate.  ^a^ Based on the instructions given for the item of occupation, some participants filled out more than one category, which explains why the sum of occupation for each quartile exceeds 100 %. | | | |
